# Obstructive Jaundice as an Initial Manifestation of Non-Hodgkin Lymphoma: Treatment Dilemma and High Mortality

**DOI:** 10.1155/2013/259642

**Published:** 2013-05-30

**Authors:** Dhara Chaudhari, Sarah Khan, Atif Saleem, Tamarro Taylor, Chakradhar Reddy, Thomas Borthwick, Mark Young

**Affiliations:** ^1^Department of Internal Medicine, East Tennessee State University, Quillen College of Medicine, P.O. Box 70622, Johnson City, TN 37614, USA; ^2^Department of Gastroenterology, East Tennessee State University, Quillen College of Medicine, Johnson City, TN 37614, USA; ^3^Department of Oncology, VA Medical Center, Quillen College of Medicine, Mountain Home, TN 37684, USA

## Abstract

*Introduction*. Non Hodgkin lymphoma (NHL) presenting with obstructive jaundice is a rare occurrence. Because of rarity of combination, it is seldom considered in differential diagnosis of patients presenting with obstructive jaundice. It is considered treatable due to the chemosensitive nature of the disease and the recent advances in chemotherapy. *Case Series*. We present a case series of 2 patients with NHL presenting with obstructive jaundice as an initial manifestation. Both patients presented with obstructive jaundice and were diagnosed by CT guided liver biopsy. One patient died of sepsis and multiorgan failure before initiating chemotherapy and the second patient did not choose to undergo chemotherapy. *Conclusion*. Biliary obstruction is a sign of poor prognosis. The diagnosis of NHL needs to be considered in patients presenting with biliary obstruction. It can be associated with high mortality and poses treatment dilemma.

## 1. Introduction

Lymphomas are malignant lymphoproliferative disorder classified into Hodgkin and non Hodgkin lymphomas (NHL). NHL is nodal and extra nodal in origin. Liver related lymphomas can be primary versus metastatic in origin. Obstructive jaundice is usually a late manifestation of NHL; however it can be a presenting feature of NHL. Incidence of obstructive jaundice as an initial presentation of NHL is only seen in 1-2% of patients [1]. Because of the rarity of this entity, careful evaluation of patients is necessary.

We present a case series of Non-Hodgkin lymphoma presenting as an obstructive jaundice and associated with high mortality.

## 2. Case 1

**Figure 1:**
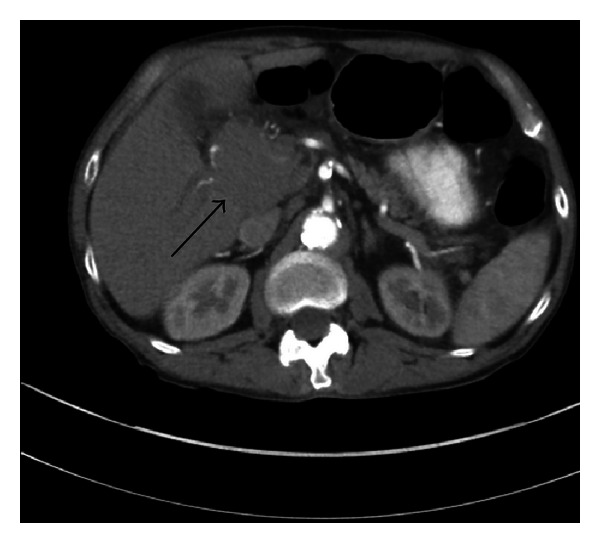
Case 1—MRI abdomen pelvis revealed 5.5 × 5.4 × 4.9 cm mass and biliary ductal dilatation from extrinsic compression (black arrow).

**Figure 2:**
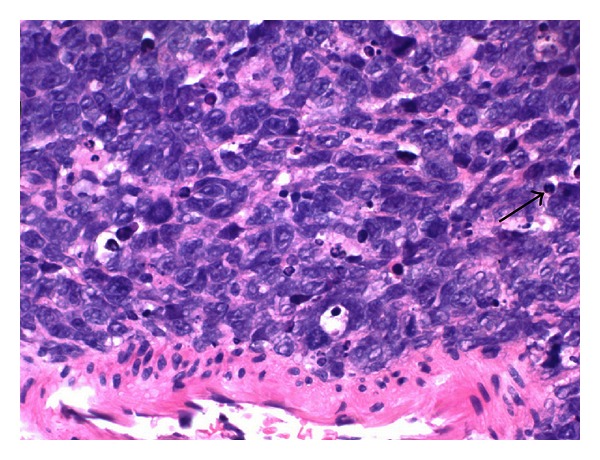
Case 1—hematoxylin and eosin stain of liver biopsy showing cells with large, hyperchromatic nuclei with scant cytoplasm and mitosis (black arrow).

**Figure 3:**
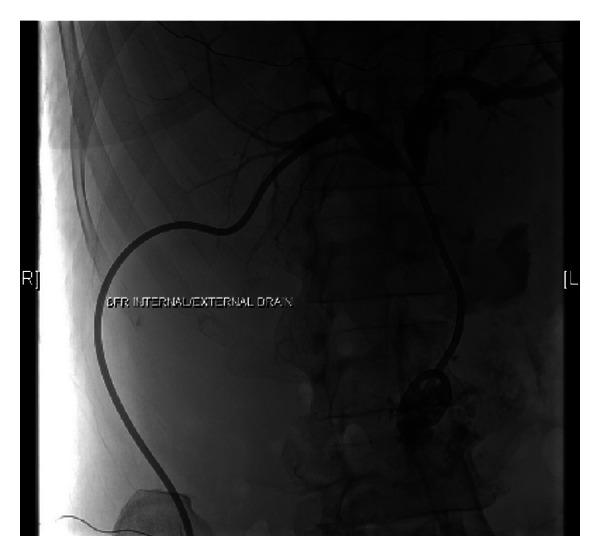
Case 1—percutaneous trans-hepatic cholangiography (PTC) with 8-French internal and external biliary drains.

A 63-year-old male with history of untreated Chronic hepatitis C and alcohol abuse was admitted to the Internal Medicine Department with 4-day history of nausea, diffuse abdominal pain, and jaundice. On physical exam he was jaundiced with scleral icterus. The abdomen was diffusely tender with guarding. Laboratory data revealed total bilirubin 16.9 mg/dL (normal: <1.0), Alkaline phosphatase 243 IU/L, aspartate transaminase (AST) 97 IU/L (normal: <35), alanine transaminase (ALT) 69 IU/L (normal: <35), alpha fetal protein: 3.0 IU/mL, lactate dehydrogenase (LDH) 267 IU/L, albumin 2.7 g/dL (normal: 3.2–4.6), total protein 5.7 g/dL (normal: 6.4–8.3), blood urea nitrogen (BUN) 14 mg/dL (normal: 8–23), creatinine 0.85 mg/dL (normal: 0.80–1.30). CBC showed white cell count (WBC): 6.3 K/*μ*L (normal: 5–10.2), hemoglobin 13.7 g/dL (normal: 13.5–17.5), and platelet 117 K/*μ*L (normal: 150–450). Liver ultrasound showed a mass-like structure around gallbladder fossa, and common bile duct (CBD) diameter was 6 mm. Computed tomography (CT) of the abdomen and pelvis showed a large irregular mass at the porta hepatis. Magnetic resonance cholangiopancreatography (MRCP) revealed 5.5 × 5.4 × 4.9 cm mass and biliary ductal dilatation from extrinsic compression (Figure 1). Pathology of fine needle biopsy of mass (Figure 2) showed diffuse large B cell lymphoma, positive for CD20, BCL2, BCL6, CD79a, and PAX5 and negative for CD3, CD5, CD10, CD23, CD138, cyclin D1, CK7, CK20, and p63. Positron emission tomography (PET) scan showed multiple liver lesions and right lower lung nodule, without involvement of mediastinal lymph nodes or peritoneum. Bone marrow biopsy was negative for lymphoma. Endoscopic retrograde cholangiography (ERC) was performed for biliary decompression but CBD could not be cannulated, and limited cholangiogram showed distal CBD stricture. Percutaneous transhepatic cholangiography (PTC) was performed with successful internal and external biliary drain placement (Figure 3). His ECOG (Eastern Cooperative Oncology Group) score was 3 and chemotherapy was planned. After few days he was readmitted with acute renal failure (BUN/Cr: 100/4.1), WBC 25.1 K/*μ*L, total bilirubin 6.4 mg/dL, intractable nausea and vomiting, and hypotension with multiorgan failure. He was found to be in septic shock. The patient died even before starting chemotherapy.

## 3. Case 2

**Figure 4:**
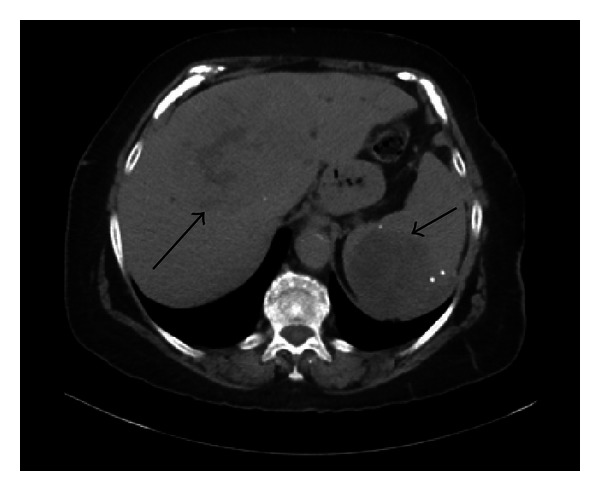
Case 2—CT scan abdomen pelvis with infiltrating ill-defined liver mass involving the portal confluence and spleen mass (black arrows).

**Figure 5:**
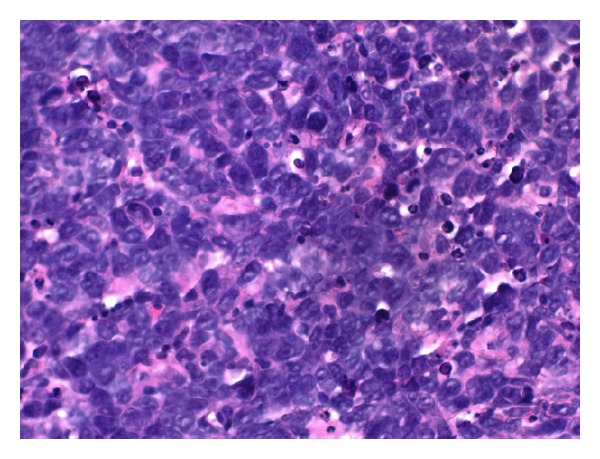
Case 2—hematoxylin and eosin stain (H&E) of liver biopsy (40X resolution) revealing diffuse large B cell lymphoma with pleomorphic cells with scant cytoplasm.

A-80-year-old nursing home female resident was admitted to internal medicine service with diffuse abdominal pain of 2-3 days duration, poor oral intake, nausea, and dark urine. PMHx was significant for dementia, hypertension, and hypothyroidism. She had cholecystectomy done at 55 years of age. She did not have history of tobacco or alcohol abuse. Physical examination revealed conjunctival icterus, confusion, tenderness in right upper quadrant, and epigastrium. No lymphadenopathy or hepatosplenomegaly was appreciated. Initial laboratory data revealed total bilirubin 12.0 mg/dL, alk phos 659 IU/L, AST 112 IU/L, ALT 86 IU/L, ammonia 35 *μ*mol/L, LDH 650 IU/L, BUN 10 mg/dL, creatinine 0.68 mg/dL, sodium 130 mmol/L, chloride 98 mmol/L, albumin 2.4 g/dL, total protein 6.4 g/dL, prothrombin time 12.7 sec, INR 1.1, partial thromboplastin time 31 sec, and WBC 15 K/*μ*L. Viral hepatitis panel, alpha-fetoprotein, and carcinoembryonic antigen were negative. CT abdomen was done revealing infiltrating liver mass involving the portal confluence with intrahepatic biliary dilatation, lesion in spleen, and absence of lymphadenopathy (Figure 4). The patient underwent percutaneous cholangiogram showing marked narrowing of biliary tree and unsuccessful attempt to insert drain. She underwent endoscopic retrograde cholangiopancreatography (ERCP) which showed distal common bile duct stricture secondary to extrinsic compression with dilated CBD up to 15 mm and intrahepatic ductal dilation. Biliary sphincterotomy was performed and plastic stent was placed for biliary decompression. CT guided liver mass core biopsy (Figure 5) reported diffuse large B cell lymphoma with stains positive for CD20 and CD79a and negative for CD10, cyclin D1, CD138, cytokeratin AE1/AE3, and S 100 protein. Her ECOG score was 4. Chemotherapy was not given because of patient's poor general condition. Patient's family decided to make her comfortable under hospice care and she succumbed within 3 months of diagnosis.

## 4. Discussion

 Diffuse large B cell lymphoma (DLBCL) is the most common form of NHL. Majority of patients with DLBCL are diagnosed in the seventh or eighth decade of life with male : female ratio [2] of 1.3 : 1. Jaundice in lymphoma patients can be secondary to direct hepatic involvement [3], extrahepatic bile duct obstruction due to enlarged lymph nodes [4], intrahepatic cholestasis, toxic hepatitis due to drug treatment, or tumor related hemolysis [5]. The most common cause of jaundice in NHL is due to extrahepatic biliary obstruction by tumor related causes [3]. Among all causes of malignant biliary obstruction, NHL accounts for 1%-2% of cases [1].

 Although jaundice is a late manifestation of NHL, it can rarely occur as an initial presentation. Rosenberg et al. in 1961 reported 1269 patients of which 159 had jaundice during disease course [5]. Three patients among those 159 had obstructive jaundice as an initial presentation [5]. The retrospective study by Ravindra et al. revealed 9 patients (6 adults and 3 children) with NHL presenting with obstructive jaundice [6]. The study by Ödemiş et al. in 2007 showed 7 patients with obstructive jaundice having NHL among 1123 patients with malignant biliary obstruction over 6-year period [1]. Because of uncommon association, NHL is seldom considered in differential diagnosis of patients presenting with obstructive jaundice. The biliary system can be compressed anywhere along the tract by lymphoma but common locations of biliary obstruction are hepatic hilum or peripancreatic head region. The elucidated mechanism of obstruction is decreased bile duct mobility in these regions making it vulnerable to pressure by neoplastic process [3].

 Although CT imaging can help identify position and burden of intra-abdominal mass, tissue biopsy is required to make definite diagnosis. Improvement in imaging methods has increased the procedural safety of image guided needle biopsy of hilar or pancreatic lesion. In patients with hepatic hilum lesion, minimally invasive methods such as CT guided mass biopsy or endoscopic brushing at ERCP can help [6]. Ravindra et al. reported a series of 9 patients with NHL, of which 2 patients had CT guided biopsy and was able to avoid surgery [6]. Endoscopic ultrasound guided fine needle aspiration (EUS-FNA) provides morphologic analysis of intra-abdominal lesions at various locations such as near gastrointestinal tract and retroperitoneal mass [7, 8]. The retrospective study by Erickson and Tertjak on 18 patients with retroperitoneal lesions undergoing EUS showed 15 patients with successful EUS-FNA with diagnosis of lymphoma in 4 patients [8]. EUS-FNA is not free of complications such as infection, hemorrhage, and risk of tumor seeding along the needle track.

 Because of infrequent presentation of NHL as an obstructive jaundice, treatment is based on few cases reported. There are no standard guidelines in the literature, which creates dilemma in management of these patients. Due to the chemosensitive nature of lymphoma and the advanced stage of the disease when present with biliary obstruction, chemotherapy is the mainstay of treatment [6, 9]. The study done by Dudgeon and Brower showed five patients with NHL and obstructive jaundice treated with chemotherapy without prior biliary decompression and resolution of jaundice within 2–59 days and had complete remission in 2 patients and partial remission in 3 patients [10]. In the retrospective analysis by Ravindra et al. on 9 patients between 1995 and 2001 with NHL and obstructive jaundice, chemotherapy was the treatment of choice [6]. Complete remission was achieved in 4 patients, partial remission in 3, and no response in one patient [6]. Among this series, five patients were treated for jaundice before chemotherapy administration but retrospectively it was considered unnecessary by authors [6].

Doxorubicin containing chemotherapy regimen is considered standard in treatment of lymphoma patients. However, since doxorubicin is being excreted through bile, its use in patients with biliary obstruction is unclear. The study by Fidias et al. showed 6 out of 7 patients in a series had initial chemotherapy without doxorubicin and one patient in a series who had stent placed with normalization of bilirubin at the time of chemotherapy initiation received doxorubicin based chemotherapy [9]. It appears safe in NHL patients with obstructive jaundice to delay the use of doxorubicin for 1-2 cycles [9]. However, fear of increasing toxicity might be a reason for hesitation to use chemotherapy in patients with abnormal liver enzymes.

The need for biliary drainage procedures for treatment of jaundice before chemotherapy administration in these patients in unclear. Many patients with obstructive jaundice have received biliary decompression with external-internal drain, stent placement, or laparotomy [10]. These treatments provide relief of obstruction and symptoms, but they do not cure underlying disease; on the other hand they could delay definitive treatment administration. Biliary stenting is recommended in patients with associated symptoms of obstruction or infectious complication of obstruction such as cholangitis [9]. Retrospective analysis by Ross et al. at MD Anderson Cancer Center of 35 lymphoma patients with obstructive jaundice undergoing ERCP/PBD between 2002 and 2008 showed normalization of bilirubin in 29 out of 33 cases and complete stricture resolution in 12 cases after biliary stent or percutaneous drain placement [11]. In our case series, both patients had biliary drainage because of severe abdominal pain in case 1 and concern of cholangitis in case 2.

Bairey et al. recently published retrospective study on 92 patients showing prevalence of early death in 7% of patients with NHL [12]. Patients in early death group had poor prognostic factors such as advanced stage of disease, old age, aggressive nature of lymphoma, poor performance status, elevated LDH, extranodal disease, and B symptoms [12]. Based on this study, the main cause of early death was sepsis, with other causes being disease progression, severe underlying disease, and gastrointestinal perforation [12]. Biliary stricture and obstructive jaundice are considered a sign of poor prognosis in lymphoma patients [6]. Based on previously reported studies by Ravindra et al. and Fidias et al., it appears that patients with initial or early jaundice presentation tend to have better survival as compared to patients with late jaundice presentation [6, 9].

 However, in our case series even though both patients had initial presentation of obstructive jaundice, both patients died within 3 months after diagnosis. Increased mortality in our case series has several possible explanations. Our patients received biliary drainage during initial course of the disease. Would that have affected their disease course by delaying early administration of chemotherapy is a question. Case 1 had developed sepsis with multiorgan failure preventing him from getting any chemotherapy. Although case 2 did not opt chemotherapy, her advanced age and poor performance status might have been the limiting factors to the beneficial effects of chemotherapy. Additionally in both cases, high grade DLBCL at the time of diagnosis with extranodal involvement, B symptoms, and bulky disease along with hepatitis C in case 1 and poor performance in case 2 could have played a part in early mortality. Early death was secondary to sepsis in case 1 and disease progression in case 2.

## 5. Conclusion

 Obstructive jaundice is a rare disease presentation of NHL. Despite rare occurrence, NHL needs to be considered in differential diagnosis while taking care of patients with obstructive jaundice. Treatment of biliary obstruction due to lymphoma is controversial regarding chemotherapy alone versus biliary drainage preceded by chemotherapy. Biliary drainage is recommended in patients with infectious complication. With increasing evidence of NHL presenting obstructive jaundice, better formulation of treatment guidelines is needed.
